# Supplementary material for: High Acceptance of COVID-19 Tracing Technologies in Taiwan: A Nationally Representative Survey Analysis
Source: Int J Environ Res Public Health. 2022 Mar 11;19(6):3323. doi: 10.3390/ijerph19063323 (PMC8954552; doi:10.3390/ijerph19063323)
Supplement: Supplementary file 1 [file ijerph-19-03323-s001.zip › ijerph-1590414-supplementary.pdf]

Supplementary Materials

Scenario Descriptions

The telecommunication network tracking scenario was described as:

"The COVID-19 pandemic has rapidly become a worldwide threat. Containing the virus' spread is essential to minimize the impact on the healthcare system, the economy, and save many lives. The Taiwanese Government might consider using phone tracking data supplied by telecommunication companies to identify and contact those who may have been exposed to people with COVID-19. This would help reduce community spread by identifying those most at risk and allowing health services to be appropriately targeted. All people using a mobile phone would be included in the project, with no possibility to opt-out. Data would be stored in an encrypted format on a secure server accessible only to the Taiwanese Government who may use the data to locate people who were violating lockdown orders and enforce them with fines and arrests where necessary. Data would also be used to inform the appropriate public health response and to contact those who might have been exposed to COVID-19, and individual quarantine orders could be made on the basis of this data."

The government App scenario was described as:

"The COVID-19 pandemic has rapidly become a worldwide threat. Containing the virus' spread is essential to minimize the impact on the healthcare system, the economy, and save many lives. The Taiwanese Government might consider using smartphone tracking data to identify and contact those who may have been exposed to people with COVID-19. This would help reduce community spread by identifying those most at risk and allowing health services to be appropriately targeted. Only people that downloaded a government App and agreed to be tracked and contacted would be included in the project. The more people that download and use this App the more effectively the Government would be able to contain the spread of COVID-19. Data would be stored in an encrypted format on a secure server accessible only to the Taiwanese Government. Data would only be used to contact those who might have been exposed to COVID-19.

And the Bluetooth (Apple and Google EN system) scenario was described as:

"The COVID-19 pandemic has rapidly become a worldwide threat. Containing the virus' spread is essential to minimize the impact on the healthcare system, the economy, and save many lives. Apple and Google have proposed adding a contact tracing capability to existing smartphones to help inform people if they have been exposed to others with COVID-19. This would help reduce community spread of COVID-19 by allowing people to voluntarily self-isolate. When two people are near each other, their phones would connect via Bluetooth. If a person is later identified as being infected, the people they have been in close proximity to are then notified without the government knowing who they are. The use of this contact tracing capability would be completely voluntary. People who are notified would not be informed who had tested positive."

Modelling

Ordinal regression of Likert responses

The following Figures displays the mean ordinal regression posterior distributions and associated Likert-style responses for items querying people's perception of COVID-19 severity and concern, government perceptions, and worldviews. Items are described in Table 2 of the main text.

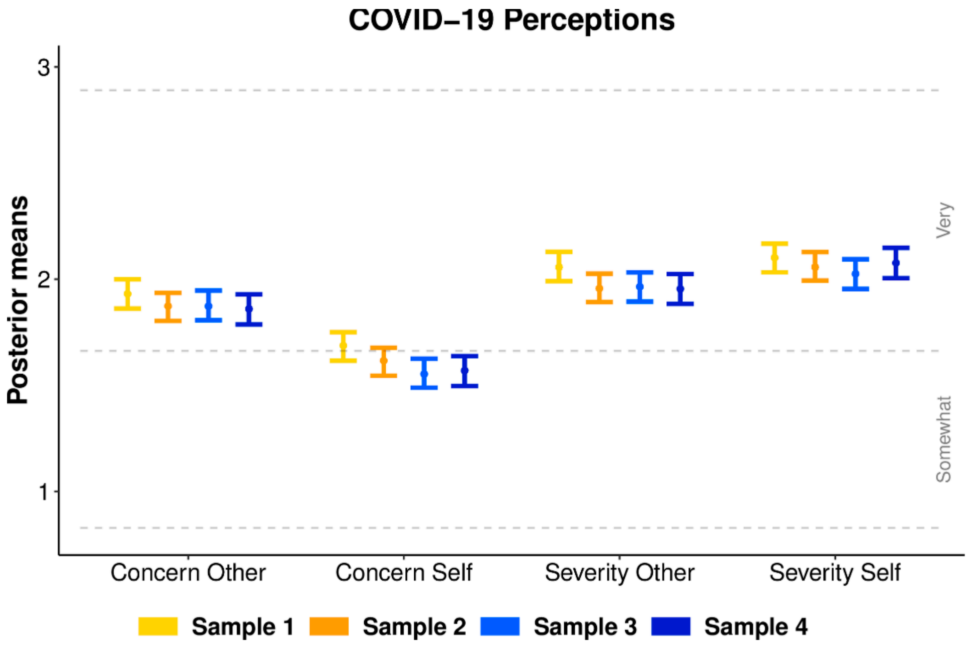

Figure S1. Ordinal regression mean posterior distributions for items assessing the perceived risk from COVID-19 in each sample. Colored error bars display the 95% highest posterior density interval. Dotted lines depict boundaries separating the latent space into ordinal responses (1 = none to 5 = extremely).

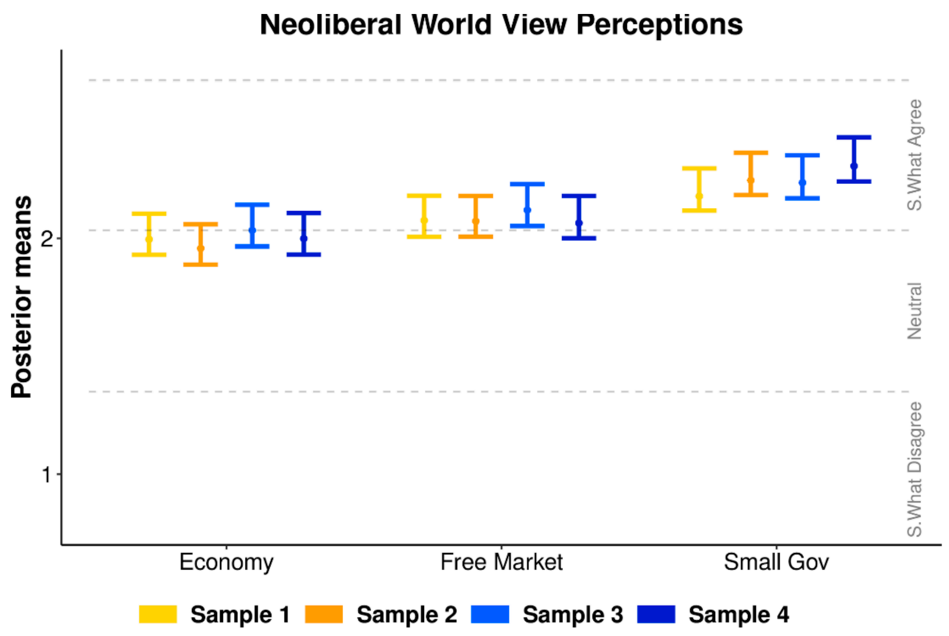

Figure S2. Ordinal regression mean posterior distributions for items assessing worldviews in each sample. Coloured error bars display the 95% highest posterior density interval. Dotted lines depict boundaries separating the latent space into ordinal responses (1 = none to 7 = extremely).

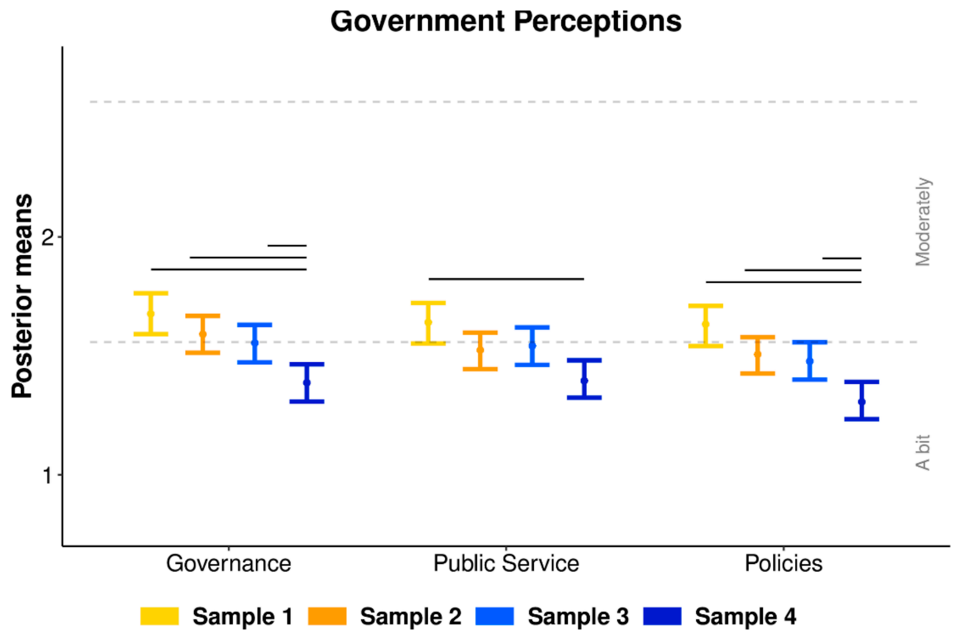

Figure S3. Ordinal regression mean posterior distributions for items assessing Government perceptions in each sample. Coloured error bars display the 95% highest posterior density interval. Dotted lines depict boundaries separating the latent space into ordinal responses (1 = none to 6 = extremely).

Bayesian generalized linear mixed effects modelling presented in the main text

Table S1. *Output of the Bayesian generalized linear mixed effects model presented in the results of the main text (with participant exclusions due to the scenario comprehension check).*

Parameter	Mean Est.	SD	95% CI Lower	95% CI Upper
Intercept	0.31	0.56	-0.81	1.34
Age	0.04	0.05	-0.06	0.14
Gender Woman	-0.05	0.09	-0.24	0.13
Education Highschool Graduate	1.03	0.45	0.15	1.94
Education University Graduate	0.94	0.44	0.09	1.83
COVID Positive Other	0.63	0.36	-0.03	1.35
COVID Lost Job	-0.05	0.17	-0.39	0.28
Gov Governance	-0.07	0.1	-0.27	0.12
Gov Policies	0.02	0.1	-0.18	0.23
Gov Services	0.06	0.07	-0.09	0.2
COVID Severity Self	0.08	0.06	-0.04	0.19
COVID Severity Other	0.02	0.06	-0.1	0.13
COVID Concern Self	0.02	0.07	-0.13	0.16
COVID Concern Other	0.06	0.07	-0.08	0.21
COVID Comply Percent	-0.1	0.05	-0.19	0
Social Distancing	0.09	0.05	-0.01	0.19
Tech Reduce Likelihood	0.57	0.09	0.4	0.74
Tech Return Activity	0.23	0.09	0.04	0.41
Tech Reduce Spread	0.24	0.09	0.06	0.41
Tech Ease To Decline	-0.05	0.05	-0.14	0.05
Tech TrustNecessary Data	0.12	0.07	-0.01	0.26
Tech Sensitive Data	-0.22	0.06	-0.33	-0.1
Tech Risk	0.07	0.06	-0.04	0.19
Tech Trust Intention	0.24	0.08	0.08	0.41
Tech Trust Privacy	0.27	0.08	0.1	0.43
Tech Trust Security	0.2	0.07	0.06	0.33
Tech Ongoing Control	0.33	0.06	0.21	0.44
WorldView Economy	0.03	0.05	-0.08	0.13
WorldView Freemarket	0.07	0.05	-0.02	0.17
WorldView Smallgov	-0.09	0.05	-0.19	0.01
Resilience	0.09	0.05	-0.01	0.19
Scenario Intercept (SD)	0.47	0.56	0.06	1.94
Rand Int. Telecommunication	0.12	0.37	-0.54	0.94
Rand Int. Bluetooth	0.13	0.37	-0.53	0.95
Rand Int. Gov App	-0.15	0.36	-0.85	0.64
Log Prob	-1552.26	4.44	-1562	-1544.6

Bayesian generalized linear mixed effects modelling: Without participant exclusions

Results were identical with and without participant exclusions based upon the scenario comprehension check, except that the model without exclusions shows an effect of small governmental neoliberal worldviews (see Figure B4 and Table B2).

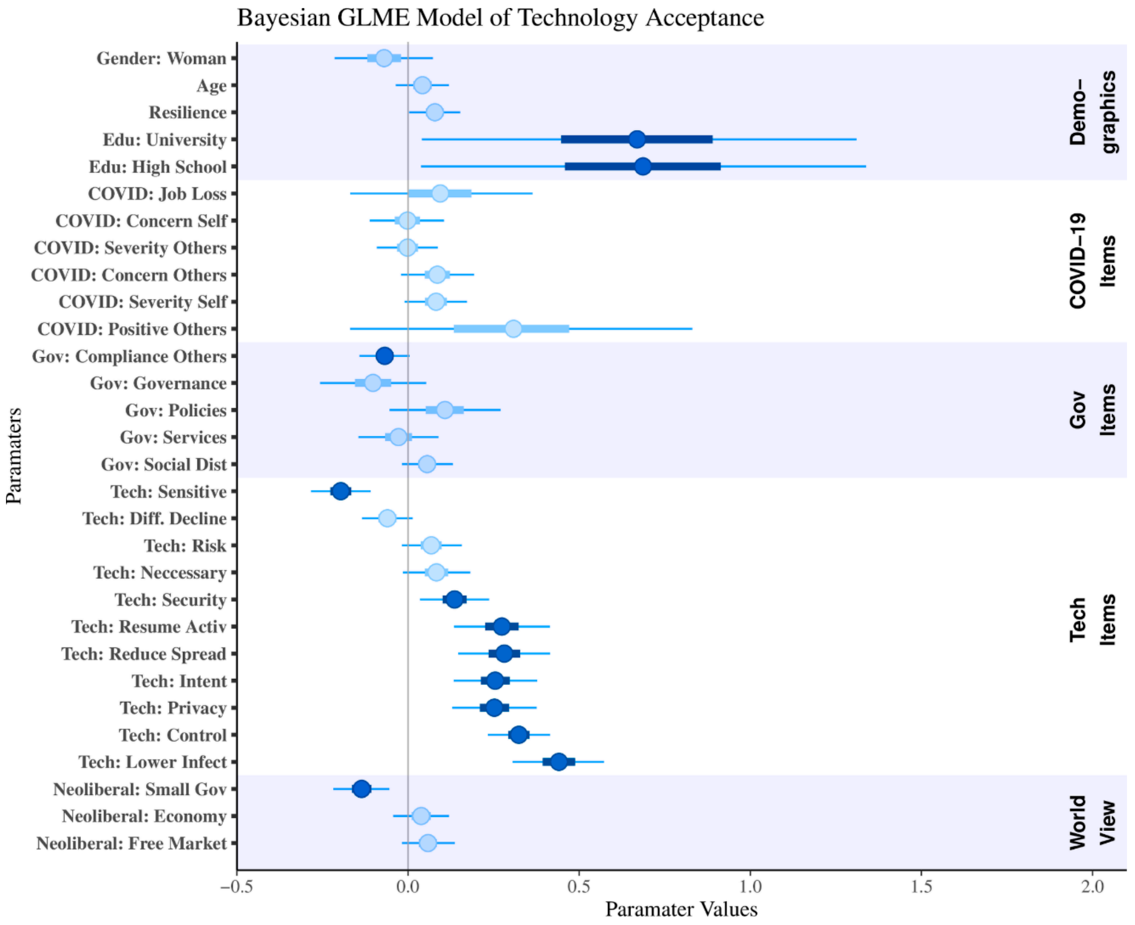

Figure S4. Bayesian generalized linear mixed effects model of tracing technology acceptance with no participant exclusions. Bars represent 50% of the parameter distribution centered on the parameter mean, tails display the 95% highest density interval. Opaque variables show instances where the posterior interval does not overlap zero.

Table S2. *Output of the Bayesian generalized linear mixed effects model without any participant exclusions due to the scenario comprehension check.*

Parameter	Mean Est.	SD	95% CI Lower	95% CI Upper
Intercept	0.53	0.41	-0.28	1.29
Age	0.04	0.04	-0.04	0.12
Gender Woman	-0.07	0.07	-0.21	0.07
Education Highschool Graduate	0.69	0.33	0.04	1.34
Education University Graduate	0.67	0.32	0.04	1.31
COVID Positive Other	0.31	0.25	-0.17	0.83
COVID Lost Job	0.09	0.14	-0.17	0.36
Gov Governance	-0.1	0.08	-0.26	0.05
Gov Policies	0.11	0.08	-0.05	0.27
Gov Services	-0.03	0.06	-0.14	0.09
COVID Severity Self	0.08	0.05	-0.01	0.17
COVID Severity Other	0	0.05	-0.09	0.09
COVID Concern Self	0	0.06	-0.11	0.11
COVID Concern Other	0.09	0.06	-0.02	0.19
COVID Comply Percent	-0.07	0.04	-0.14	0
Social Distancing	0.06	0.04	-0.02	0.13
Tech Reduce Likelihood	0.44	0.07	0.31	0.57
Tech Return Activity	0.27	0.07	0.13	0.41
Tech Reduce Spread	0.28	0.07	0.15	0.41
Tech Ease To Decline	-0.06	0.04	-0.13	0.01
Tech Trust Necessary Data	0.08	0.05	-0.01	0.18
Tech Sensitive Data	-0.2	0.04	-0.28	-0.11
Tech Risk	0.07	0.05	-0.02	0.16
Tech Trust Intention	0.25	0.06	0.13	0.38
Tech Trust Privacy	0.25	0.06	0.13	0.38
Tech Trust Security	0.14	0.05	0.03	0.24
Tech Ongoing Control	0.32	0.05	0.23	0.42
WorldView Economy	0.04	0.04	-0.04	0.12
WorldView Freemarket	0.06	0.04	-0.02	0.14
WorldView Smallgov	-0.14	0.04	-0.22	-0.05
Resilience	0.08	0.04	0	0.15
Scenario Intercept (SD)	0.33	0.44	0.03	1.49
Rand Int. Telecommunication	0.07	0.27	-0.41	0.64
Rand Int. Bluetooth	0.07	0.26	-0.41	0.65
Rand Int. Gov App	-0.1	0.26	-0.61	0.44
Log Prob	-2509.38	4.47	-2519.11	-2501.67
